# On the Prevalence of Addicted or Problematic Gaming in Finland

**DOI:** 10.1016/j.abrep.2019.100225

**Published:** 2019-11-06

**Authors:** Veli-Matti Karhulahti, Raine Koskimaa

**Affiliations:** a University of Jyväskylä, Finland; b University of Turku, Finland

**Keywords:** Gaming disorder, Internet gaming disorder, Prevalence, Finland

## Abstract

We bring attention to a notable unreported “gaming addiction” or “problem gaming” prevalence data set that adds significantly to both the DSM-5′s call for further Internet Gaming Disorder research as well as many other scientific encouragements for more wide-ranging reports on the topic.

## Highlights

• A dataset from Finland in 2015 (systematic random sampling N = 4511) suggests an (unweighted) prevalence rate of certain “addicted gaming” (videogame play) to be 0.6% among local gamers and 0.03% among the whole population.
• The implied prevalence of certain “problematic gaming” (videogame play) climbs to 1.4% among local gamers and 0.6% among the whole population.
• Of those “addicted” individuals to whom videogame play was a problem “almost always,” eight reported their hours of play during a week (72, 30, 10, 7, 4, 1, 1, 1), which indicate that “addicted gaming,” if understood as excessive play, might not be optimal for describing such problems.

Videogame play is entering the addiction discourse via both the *Diagnostic and Statistical Manual of Mental Disorders* (DSM-5) (American Psychiatric Association, 2013) and the *International Classification of Diseases* (ICD-11) (World Health Organization, 2019), the former of which classifies the issue as a condition for further study under the label “Internet Gaming Disorder” (IGD). This has spawned plenty of non-academic (for a review see King, 2018) as well as academic criticism, the latter of which is aptly summarized by a recent paper from 37 scholars (van Rooij et al., 2018).

One of the key criticisms (re)raised by the multi-authored paper is the imbalance of related data and its lack of cultural width. As an example, the scholars reference a data set from Singapore that has been used in upward of 19 publications, some of which have been problematized for their research practices (see Przybylski and Weinstein, 2019, Ferguson and Wang, 2019). Ultimately, the scholars conclude that in “countries where excessive gaming is perceived to be a problem, it may be more appropriate to seek solutions on a domestic level [which] would enable cultural and context-specific considerations” (van Rooij et al., 2018, 5)

Accordingly, we would like to bring attention to a notable unreported data set that adds to both the APA’s (American Psychiatric Association, 2013) call for further IGD research as well as the encouragement by van Rooij et al. (2018) for more wide-ranging reports on the topic.

In 2015, Finland’s *National Institute for Health and Welfare* (THL), aided by the *Population Register Centre*, conducted a representative survey related to local gambling practices (Salonen & Raisamo, 2015). While the objective of the survey (systematic random sampling N = 4511) was to chart gambling issues, it also included five questions related to non-gambling gaming:

1. Have you played video, computer, or mobile games in the past 12 months?
2. How often have you played these games?
3. How many hours have you played in the last 7 days?
4. How many hours have you played in the last 30 days?
5. How often have you felt that playing video, computer, or mobile games could be a problem for you in the past 12 months?

These questions were not discussed in THL’s final report. While all the survey data was donated to the *Finnish Social Science Data Archive* for open use, to our knowledge, no publication has drawn from the mentioned five items in the sample. We thus provide a summary of the local gaming figures, including derived Finnish prevalence rates of the related “gaming addiction” or “problem gaming.” Clearly, a one-item measure of gaming problems (Question 5) is suboptimal for producing reliable scientific evidence in many ways; however, with this limitation considered, rather than claiming the above as reliable scientific evidence for “gaming addiction” or “problem gaming” in general, we present the results as local findings that may be useful for other scholars and future research as a distinct regional instance.

Altogether, 2009 respondents (45 percent of all respondents) reported having played videogames in the past 12 months. These gamers answered the fifth question with the below distribution:

a) Never (1808)
b) Sometimes (171)
c) Often (16)
d) Almost always (13)

In the present Finnish data, those who reported having problems with gaming “almost always” (d) would add up to an (unweighted) prevalence of 0.6 percent among gamers and 0.03 percent among the whole population. If those who reported having problems with gaming “often” are included (c + d), the rate climbs to 1.4 percent among gamers and 0.6 percent among the whole population.

One way to accommodate these figures to the current discourse would be to call the first group “addicted gamers” because they report constant problems, and the second group “problem gamers” because they report frequent problems. A similar (not the same) logic has been used earlier, for instance, in a large-scale nationally representative study on Norwegian gamers (N = 3389), where those who reported issues “sometimes” on four items (relapse, withdrawal, conflict, problems) were classified as “addicted gamers” (1.4 percent) and those who reported issues “sometimes” on two or three items were classified as “problem gamers” (7.3 percent) (Wittek et al., 2016, Lemmens et al., 2009).

Of note, and with reference to the above, the prevalence rates related to (internet) gaming disorder or pathological gaming (or “gaming addiction” or “problem gaming”) vary greatly across studies. This variation is arguably a result of methodological differences. One comprehensive meta-analysis (Ferguson, Coulson, & Barnett, 2011) found the general prevalence of the phenomenon to be approximately 6.0 percent, whereas with more precise measures the rate dropped to 3.1 percent. Later studies have found the actual prevalence rates to be even lower (e.g. Przybylski, Weinstein, & Murayama, 2016; cf. Männikkö, Billieux, & Kääriäinen, 2015), and our data, regardless of its limitations, falls somewhat accurately within these later figures.

In order to better understand those gamers in our sample to whom videogames are a problem “almost always,” we took a closer look at their responses. Of this subsample (N = 13), eight individuals reported their time spent on playing during the week, with the following respective gaming hours: 72, 30, 10, 7, 4, 1, 1, and 1. The numbers indicate that what people consider a “problem” with gaming that occurs “almost always” may not necessarily reflect what scholars and others typically consider “problem gaming” let alone “addicted gaming,” especially the latter and its adverse consequences being often associated to large amounts of gaming hours. To sum, the term “addictive gaming” might not be optimal for describing those who consider gaming a constant personal problem.

## Declaration of Competing Interest

The authors declare that they have no known competing financial interests or personal relationships that could have appeared to influence the work reported in this paper.
